# Predicting implementation: comparing validated measures of intention and assessing the role of motivation when designing behavioral interventions

**DOI:** 10.1186/s43058-020-00050-4

**Published:** 2020-09-28

**Authors:** Jessica Fishman, Viktor Lushin, David S. Mandell

**Affiliations:** 1 Department of Psychiatry, Perelman School of Medicine, University of Pennsylvania, Philadelphia, USA; 2 Annenberg School, University of Pennsylvania, Philadelphia, USA; 3 Leonard Davis Institute of Health Economics, Perelman School of Medicine, University of Pennsylvania, Philadelphia, USA

**Keywords:** Behavioral intentions, Motivation, Methods, Measurement, Questionnaire, Predicting EBP implementation, Causal models, Theory

## Abstract

**Background:**

Behavioral intention (which captures one’s level of motivation to perform a behavior) is considered a causal and proximal mechanism influencing the use of evidence-based practice (EBP). Implementation studies have measured intention differently, and it is unclear which is most predictive. Some use items referring to “evidence-based practice” in general, whereas others refer to a specific EBP. There are also unresolved debates about whether item stems should be worded “I intend to,” “I will,” or “How likely are you to” and if a single-item measure can suffice. Using each stem to refer to either a specific EBP or to “evidence-based practice,” this study compares the ability of these commonly used measures to predict future EBP implementation. The predictive validity is important for causal model testing and the development of effective implementation strategies.

**Methods:**

A longitudinal study enrolled 70 teachers to track their use of two EBPs and compare the predictive validity of six different items measuring teachers’ intention. The measures differ by whether an item refers to a specific EBP, or to “evidence-based practices” in general, and whether the stem is worded in one of the three ways: “I intend to,” “I will,” or “How likely are you to.” For each item, linear regressions estimated the variance in future behavior explained. We also compared the predictive validity of a single item versus an aggregate of items by inter-correlating the items using different stems and estimating the explained variance in EBP implementation.

**Results:**

Depending on the EBP and how intention was measured, the explained variance in implementation ranged from 3.5 to 29.0%. Measures that referred to a specific EBP, rather than “evidence-based practices” in general, accounted for more variance in implementation (e.g., 29.0% vs. 8.6%, and 11.3% vs. 3.5%). The predictive validity varied depending on whether stems were worded “I intend to,” “I will,” or “How likely are you to.”

**Conclusions:**

The observed strength of the association between intentions and EBP use will depend on how intention is measured. The association was much stronger if an item referred to a specific EBP, rather than EBP in general. To predict implementation, the results support using an aggregate of two or three intention items that refer to the specific EBP. An even more pragmatic measure of intention consisting of a single item can also predict implementation. As discussed, the relationship will also vary depending on the EBP, which has direct implications for causal model testing and the design of implementation strategies.

Contributions to the literature

- Implementation science has increasingly emphasized the need for standardized measures that can predict EBP implementation.
- Research has suggested that behavioral intention is a causal and proximal mediator (or mechanism) of behavior, including whether practitioners implement an EBP.
- Because implementation studies have measured intention differently, we compare how well six popular measures predict EBP usage.
- We found that measures of intention predict EBP usage and that the strength of this association varies greatly depending on how intention is measured.
- Measures that refer to a specific EBP are more predictive than those that refer in general to “evidence-based practices.” An aggregate of two or three items measuring intention, or even a single item alone, can predict EBP implementation.
- Prediction also depends on the EBP, which has direct implications for causal model testing and the development of effective implementation strategies.

## Background

Health care, education, and other fields are making substantial efforts to increase the use of evidence-based practice (EBP). The success of these efforts may depend partially on organizational factors and factors external to the organization [1–5]. Ultimately, however, implementation depends on whether individual practitioners change their behavior and start using an EBP [1–5]. Even within the same organization, under the same leadership, some practitioners will use an EBP, while others will not [4, 5]. It is therefore important for implementation science to influence practitioner behavior.

Within implementation science, there is growing interest in studying the influence of behavioral intention on behavior [5–8]. Williams describes intention as an “important antecedent” to the adoption of a practice or treatment [8]. Moullin et al. argue that “intentions are a critical determinant of implementation behavior” [7]. We have also made this case when measuring intentions to implement EBP [5, 6, 9, 10]. These claims reflect a large literature in social psychology documenting the relationship between behavior and behavioral intention, where the latter is defined as one’s level of motivation or commitment to perform the behavior [11, 12]. Applied to implementation science, when practitioners strongly intend to start using an EBP (or “best practice”), they are inclined to exert substantial effort to change their behavior accordingly [5, 6].

Intention plays an important role in validated models of behavior from psychology (e.g., the Theory of Reasoned Action [11], Theory of Planned Behavior [13], Social Cognitive Theory [14], Unified Theory of Behavior [15], and the Integrated Model [16]). Much experimental and observational evidence generated over several decades suggests that behavioral intentions are malleable and that changes in the strength of intention often are followed by changes in behavior [12, 17, 18]. Intention is considered to be the most proximal mediator of behavior since it is the construct most likely to predict voluntary behavior [12, 17–21]. For example, studies find that intention explains more variance in behavior than attitudes, norms, self-efficacy, perceptions of risk and severity [19–22], personality factors, or socio-demographic variables [22–24]. As another example, when there is a conflict between what people want to do and what they intend to do (because they feel they should), intention has a greater predictive validity [25]. This large literature in psychology studying the relationship between behavior and intention is relevant given that implementation science seeks to understand and change adult behavior within organizational contexts [5, 6].

Although implementation scientists have become increasingly interested in studying intention, they lack consensus on how intentions should be measured, resulting in fundamentally different approaches. The absence of standardized measures for a commonly used construct, such as intention, creates several problems for implementation research. Broadly speaking, the lack of standardization “threaten[s] the strength of implementation science’s developing knowledge base” [26]. Without standardized measurement, it is difficult for implementation science to develop a common scientific language and compare or pool findings across studies [26, 27].

In some studies, measures of intention refer to a specific EBP of interest. One such study developed items separately for each of six best practices recommended for diabetes management [1]. For example, one item measured how strongly providers intend to give their patients weight management advice, and another item measured how strongly they intend to examine patients’ foot circulation and sensation [1]. Other studies measure intention with items that refer generally to evidence-based practices [8, 28, 29]. For example, Williams developed a measure of intention that includes the item “I intend to use an evidence-based treatment” [8]. Aarons developed an item asking, “how likely would you be to adopt” evidence-based practices, defined as any “method or intervention that was new to you” [29, 30]. Menezes et al. developed a 35-item measure of intention for use within organizations that includes items asking about “a task you have never performed before” [28]. In their questionnaire manual for health services researchers, Francis et al. recommend that the “methods used to measure intentions should be guided by researchers’ judgments about which types of questions seem to make sense” [31].

However, as Moullin et al. recently stressed, implementation science needs to develop a standardized measure of intention with predictive validity [7]. Several psychometric properties of measures can be evaluated, but predictive validity—which refers to the degree to which measurement correlates with a variable that is assessed at a later point—is often considered to be the most important [32, 33]. Predictive validity can be used to evaluate theorized relationships between constructs in causal models [32–35] and inform the development of effective implementation strategies.

### Specific study goals

The present study compares the predictive validity of different approaches used to measure intention to implement an EBP, and for this purpose, it includes three specific goals. The first goal is to compare the predictive validity of measures referring generally to “evidence-based practices” with those specifying a particular EBP. An item referring generally to “evidence-based practices” could be used conveniently when studying any type of implementation, with no need to adapt a measure for a specific EBP. On the other hand, measures referring broadly to a collection of EBPs may demonstrate weaker predictive validity [5, 6]. We hypothesized that measures referencing a specific EBP would demonstrate stronger predictive validity than measures referring to “evidence-based practices.”

Our second goal was to compare the predictive validity of three different item stems that have been used widely in social psychology research to measure intention [17–19]. These stems are worded “I intend to,” “I will,” or “How likely are you to.” Each stem was designed to be adapted to any behavior of interest, and each has been used to predict many types of behavior [12, 17–21]. The relative merits of these three stems are unclear, leading to debates about which is superior, and whether they are interchangeable [21, 36–38]. Meta-analytic comparisons have generated mixed results and did not include studies that administered all items to the same respondents [21, 37, 38]. The few studies that administered multiple types of items to the same respondents also had mixed results [38, 39]. No known studies have compared predictive validity when studying EBP implementation.

Our third goal was to test if the predictive validity of the measure depends on the EBP. We studied two EBPs, one of which is more complicated in that it requires a series of interactive tasks and is more resource-dependent than the other. Prior research suggests that intentions are more likely to be translated into action when the behavior of interest is less complicated [12, 40]. In turn, we hypothesized that intention would better predict the performance of a less complicated EBP.

This study was conducted as part of a larger line of research investigating how to increase EBP use in public schools. Teachers who implement EBP can have a substantially positive impact on youth, including the most vulnerable youth, such as those with autism, who receive most of their interventions at school [41, 42]. The current study focuses on teachers’ use of visual schedules and discrete trial training (DT) in classrooms serving students with autism. When these EBPs are used by teachers, children show improved emotional, social, and academic outcomes [41–45]. These two EBPs are used as examples of EBPs that improve student outcomes but are not implemented frequently [41–45].

## Methods

### Study design, sample, and study site

We conducted a longitudinal cohort study in partnership with the School District of Philadelphia. It was approved by the Institutional Review Boards of the School District and the University of Pennsylvania. Based on our prior studies, we estimated that we could recruit 75% of eligible teachers [43–45]. We therefore proposed a sample of 70 teachers from a total of 91 teachers of the district’s kindergarten-through-second-grade autism support classrooms. All 91 teachers were invited in the first 3 weeks of the academic year during the first professional development day of the year or during an in-school visit. We contacted each teacher until we received a response or until we reached the enrollment goal of 70 teachers.

### Two examples of EBP

The National Standards Report of evidence-based practices for children with autism identifies visual schedules and discrete trial training (DT) as core components of many comprehensive intervention models [41–44]. Teachers in our sample had received didactic training and in-classroom coaching sessions on both EBPs and possessed at least the minimum skills needed to use each [43, 44]. We selected these two EBPs for the present study because they have different levels of complexity, as explained below, which was expected to influence their implementation.

*Visual schedules* require teachers to prominently post a guide that illustrates, for each child, the order of school activities. In this study, all teachers were given the materials needed to create the visual schedules. When the needed resources are made available, this practice is relatively easy to implement. Teachers are expected to post the schedule, and during each transition, they should tell students to check it so that students learn which activity is next and increase their independence.

*Discrete trail training* (*DT*) requires teachers to work with each student individually to improve academic or pre-academic skills. This EBP requires a series of interactions between the teacher and a single student, plus the teacher is responsible for intensive data collection [41–44]. In addition to being more complicated than visual schedules, DT is highly resource-dependent because the prolonged interactions between a teacher and a student require dedicated space and materials, uninterrupted time, and additional classroom staff to supervise the other students.

### Baseline survey questionnaire

After teachers received didactic training and in-classroom coaching for each EBP, they completed a self-administered survey questionnaire measuring the strength of their intention to use visual schedules and DT. We administered the questionnaire afterwards to ensure that teachers were aware of why and how a teacher can use visual schedules and DT, which in turn allows them to give meaningful responses to the questionnaire. To avoid burdening teachers earlier in the school year when they are especially busy, the questionnaire was administered about 2 months after the training. We also expected that data collected early in the year would be less generalizable because teacher behavior (and presumably their intention) does not reflect their usual behavior [43–45].

#### Item stems used to measure intention

As noted above, the questionnaire items measuring intention included three stems used commonly in social psychology that are meant to be adapted to any behavior of interest [12, 16]. The three stems are worded:

*I intend to* [perform the behavior].

*I will* [perform the behavior].

*How likely are you* to [perform the behavior]?

In social psychology, these stems have been paired with standardized response options that use scales ranging from a strong intention to not engage in the behavior, through uncertainty, and to a strong intention to engage in the behavior [12, 16]. With the first two above stems (worded as “I intend to” and “I will”), the scaled response options ranged from 1 to 7, anchored descriptively with “strongly disagree” to “strongly agree.” The item stem worded, “How likely are you to” included scaled response options ranging again from 1 to 7 that were anchored descriptively with “extremely unlikely” to “extremely likely.” Each scale’s mid-point anchor was neutral.

#### Items reference either a specific EBP or EBP in general

In social psychology, the recommended approach for measuring intention requires that an item refers to a relatively specific behavior of interest [12, 19]. The recommended approach defines the frequency and duration of the behavior involved. In line with this approach, the survey questionnaire told the respondents to think about the next 3 months and defined one EBP as “running discrete trial training (DT) at least 3 days a week.” The other EBP was defined as “using individualized student schedules during each transition.” The questionnaire included three items measuring intention that referred specifically to DT while varying the item stem. By varying the item stem again, another set of three intention items referred to visual schedules.

Because some implementation science studies rely on items that refer to EBPs in general, the questionnaire also adapted the three intention item stems to ask about a teacher’s intention to “use evidence-based practices.” These items were worded, “I intend to use evidence-based practices,” “I will use evidence-based practices,” and “How likely are you to use evidence-based practices?” The response options were the same as those described above (and illustrated in Additional file 1).

#### Items measuring socio-demographic variables

To characterize the sample for comparisons across studies, the baseline questionnaire asked teachers to report socio-demographic information, including gender, age, race, and ethnicity. Teachers also reported if they had received specialized training for teaching children with autism in prior years, and the number of years spent teaching special education.

### Measuring EBP use at follow-up as the outcome

Throughout one academic year, using validated methods [5, 43, 44], teachers reported their use of visual schedules and DT. For each student in their class, teachers recorded a log if and when they used each EBP. We asked teachers to update their log at the end of each day. During monthly visits, a study team member would review the log with the teacher for the prior week. If the log was missing information for that week, the teacher completed the log using work products (such as completed lessons) and recall. The present study analyzed the log data from the first monthly visit.

The log included the name of each student, day of the week, and a column for each EBP that allowed them to check a box to select a response option indicating how frequently they used each practice with each student that day. For example, teachers specified the frequency with which they used visual schedules during the last week (see Additional file 1). For each student, the teacher selected a scaled response, where the descriptive anchors ranged from 0 = “never” to 4 = “at every transition.” Teachers also reported how often they conducted DT. For each student, the teacher selected from a scale ranging from 0 = “less than once a week” to 4 = “twice a day.” We computed means across students to provide the average weekly frequency with which teachers used each EBP with the students in their class.

### Analytic strategy

There were no missing data for any of the variables used in the following analyses. For each EBP, we compared the predictive utility of three intention items (each with a different stem) that referred specifically to each practice, versus three intention items (each with a different stem) that mentioned “evidence-based practice” in general. Using a set of linear regression models, we analyzed overall explained variance in use of each EBP, as explained by each of the three intention items that either refer generally to “evidence-based practice,” or that specify the EBP and by combinations of these items (see Tables 2 and 3, right column). Then, for each EBP, we tested whether the predictive validity of an item depended on which stem was used. To do this, we compared the bivariate correlations between each intention item and use of each EBP (see Tables 2 and 3, middle column). We used these coefficients to examine which measures of intention (predictor variables) are more strongly associated with the use of each EBP (outcome variables). The interpretation of each coefficient is the number of standard deviations the outcome variable increases, on average, with one standard deviation increase of the predictor variable [46].

We also explored whether using a composite of two or three intention items accounted for more variance in the use of the EBP than using a single item. We examined the correlations among the triad of intention items that referred to visual schedules, DT specifically and “evidence-based practices” generally. Across the triads, we also derived overall proportions of outcome variance explained by each individual intention item.

## Results

### Sample characteristics

Table 1 displays the study’s sample characteristics. Teachers self-identified predominantly as white females with an average of approximately 7 years of experience teaching special education. Their mean strength of intention to use either EBP varied depending on the item used to measure it. When referring to “evidence-based practices” in general, the strength of intention ranged from 6.13 (SD = 1.56) to 6.60 (SD = 0.69), depending on how the item stem was worded. The means for the three items that referred to visual schedules ranged from 5.48 (SD = 2.13) to 6.07 (SD = 1.66). The means for three intention items that referred to DT ranged from 6.13 (SD = 0.93) to 6.40 (SD = 0.81).

**Table 1:** Descriptive statistics for the sample’s EBP use, intention, and socio-demographics (*N* = 70)

Variable	*N* (%)	M (SD)
**Frequency of EBP use** (self-reported with a 0–4 scale)		
Visual schedule use		1.93 (1.58)
Performance of DT		0.76 (0.9)
**Intention items referring to a specific EBP** (1–7 scale)		
I Intend to use visual schedules.		6.07 (1.66)
I will use visual schedules.		5.98 (1.7)
How likely are you to use visual schedules?		5.48 (2.13)
I Intend to use DT.		6.40 (0.81)
I will use DT.		6.38 (0.81)
How likely are you to use DT?		6.13 (0.93)
**Intention items referring to EBP in general** (1–7 scale)		
I Intend to use EBPs.		6.60 (0.69)
I will use EBPs.		6.60 (0.58)
How likely are you to use EBPs?		6.13 (1.56)
**Socio-demographics**		
Age (in years)		35.28 (10.52)
Experience teaching special education (in years)		6.92 (5.93)
Specialized training working with ASD children		
Yes	*24* (34)	
No	*41* (59)	
Not provided	*5* (7)	
Gender		
Female	*62* (88)	
Male	*5* (8)	
Not provided	*3* (4)	
Race		
White	*47* (68)	
Black	*9* (13)	
Others	*7* (10)	
Not provided	*7* (10)	
Ethnicity		
Hispanic	*3* (4)	
Non-Hispanic	*66* (94)	
Not provided	*1* (1)	

Teachers were more likely to use visual schedules than DT. The mean frequency with which they used visual schedules was 1.93, a score that falls between “few transitions” and “some transitions” on the 0 to 4 response option scale. The mean score for performing DT was 0.76, which corresponds to about “less than once per week.”

### Comparing the explained variance in behavior for general versus specific intention items

Together, the three intention items referring generally to “evidence-based practices” explained 8.6% of the overall variance in the use of visual schedules and 3.5% of the variance in the use of DT. Meanwhile, the three intention items that referred specifically to visual schedule use together explained 29.0% of the variance in the use of visual schedules. Together, the three intention items specifying DT use explained 11.3% of the variance in DT use.

### Correlating behavior with general intention items and specific intention items

As presented in Tables 2 and 3, the use of visual schedules was correlated with each of the intention items that mentioned this EBP: “I intend to” use visual schedules (*r* = 0.6, *p* < 0.01), “I will use” visual schedules (*r* = 0.6, *p* < 0.01), and “How likely are you to use” visual schedules (*r* = 0.6, *p* < 0.01). The use of visual schedules was not correlated with any of the three general intention items (in each case, *p >* 0.05). Teachers’ performance of DT was not correlated with any of the three specific or three general intention items (in each case, *p* > 0.05).

**Table 2:** Visual schedule use predicted by measures of intentions

Intention items	Correlation with the teacher’s future visual schedule use	Explained variance (*R*^2^) in teacher’s future visual schedule use
I intend to use EBPs.	0.182	0.033
I will use visual EBPs.	0.196	0.038
How likely are you to use EBPs?	0.244	0.060
I intend to use visual schedules. (*A*)	0.552*	0.266
I will use visual schedules. (*B*)	0.553*	0.277
How likely are you to use visual schedules? (*C*)	0.467*	0.202
*A*, *B*, and *C* (combined)		0.290
*A* and *B* (combined)		0.277

*p < 0.01

**Table 3:** Discrete trial training (DT) use predicted by measures of intention

Intention items	Correlation with the teacher’s future DT use	Explained variance (*R*^2^) in teacher’s future DT use
I intend to use EBPs.	0.182	0.030
I will use EBPs.	0.185	0.030
How likely are you to use EBPs?	0.057	0.003
I intend to use DT. (*A*)	0.271	0.052
I will use DT. (*B*)	0.177	0.032
How likely are you to Use DT? (*C*)	0.104	0.014
*A*, *B*, and *C* (combined)		0.113
*A* and *B* (combined)		0.084

### Correlating behavior with a single item versus an aggregate

For the intention items referring specifically to visual schedules, those with the “I intend to” and “I will use” stems were highly correlated (*r* = 0.97, *p* < 0.01). Both items were also correlated with the item asking “How likely are you to” use visual schedules (for both dyads, *r* = 0.5, *p* < 0.01). Likewise, for the intention items referring specifically to DT, those with the “I intend to use” and “I will use” stems were highly correlated (*r* = 0.9, *p* < 0.01). Both were also correlated with the item asking “How likely are you to” use DT (for both dyads, *r* = 0.5, *p* < 0.01).

As presented in Tables 2 and 3, items that referred specifically to teachers’ intentions to use visual schedules (“I intend to use,” “I will use,” and “How likely are you to”) explained 27%, 28%, and 20%, respectively, of the variance in visual schedule use. The *R*^2^ statistics of the three items were not statistically significantly different from each other, or with the *R*^2^ of the three items combined. The variance explained by the two “top performing” items together (28%) did not significantly differ from the variance explained by the three items together (29%).

Items that referred specifically to teachers’ intentions to use DT explained 5%, 3%, and 1% of the variance in DT use (Table 3). These differences were not statistically significant. For DT use, variance explained by two “top-performing” items together (8%) did not statistically significantly differ from the variance explained by three items together (11%). Both aggregates explained significantly more variance in DT use than any individual intention items referring to DT use.

## Discussion

Because the predictive validity of behavioral intention varies widely depending on how the construct is measured, the results presented have implications for implementation science’s search for a standardized measure. The results also demonstrate how predictive validity varies depending on the EBP, which has implications for the design of implementation strategies.

The following five observations elaborate on these implications:

1. Measures of intention that refer generally to “evidence-based practice” have much less predictive validity than those that refer to a specific EBP. The stronger predictive validity of items referring to a specific EBP is consistent with the “gold standard” approach in traditional social psychology [16–19]. Currently, however, many implementation studies use the alternative approach which refers generally to the collection of EBPs [7, 8, 28–31]. Predictive validity may be diminished when an item refers to “evidence-based practice” if the strength of intention to use an EBP may vary depending on the EBP. In turn, individuals may face confusion about which specific EBP they should keep in mind when a questionnaire item requires one response option. Indeed, for the present study, and many others [5, 6], the strength of intention does vary depending on the EBP.

2. Implementation can be predicted by a single-item, highly pragmatic measure of intention. Moullin et al. stress that “there is a need for the development and testing of pragmatic measures of providers intentions” [11]. A pragmatic measure should be applicable to studies of different EBPs [30, 47]. As illustrated by this and other studies [5, 6], the items stems are easily adapted to any EBP, meaning they offer a measurement approach that is standardized but also flexible in a useful way.

Pragmatic measures are also brief and easy to complete [47], but several instruments used commonly in implementation science studies include dozens of items per construct, making them impractical to use in many research settings [27]. Brief instruments are more likely to minimize respondent errors due to fatigue and carelessness [30, 47]. The current study suggests that intention to implement an EBP can be measured using just one item. For reasons discussed below, the “I intend” stem may be the most promising single-item measure of intention.

3. Although implementation can be predicted by a single-item, for some EBPs, predictive validity can increase when two or three items are aggregated. A substantial proportion of variance in EBP use was explained by a single-item measure of intention, with little additional value added by one or two more items. Among the items that referred specifically to visual schedules, those with the “I intend to” and “I will” stems each demonstrated strong predictive validity, and the difference in their ability to predict the future use of visual schedules was not statistically significant. These two items were highly correlated, and they may have measured the same latent construct. Each item explained nearly as much outcome variance as the aggregate measures that combined two or three items specifically referring to visual schedules. In this case, it would be useful to minimize respondent burden by measuring intention with just one item. The “I intend” stem may be preferable given it has high predictive utility and the strongest face validity with the intention construct.

In some cases, predictive validity may be substantially increased by measuring intention using an aggregate of two or three items. For example, the combination of two or three intention items referencing DT explained a significantly greater proportion of variance in DT use than any individual intention item. Yet, even these composites did not account for a large amount of variance, which is instructive, as discussed below.

4. The degree to which intentions can predict EBP use will depend on the EBP.

Presseau et al. write that “behavioral theory is often tested on one behavior in isolation from other behaviors” [1]. By studying more than one EBP, we demonstrated that the predictive validity of intention can vary even among practitioners who work in the same organization and implementation context. (This variation is consistent with models of behavior from social psychology, such as the Theory of Planned Behavior.)

The study included two very different EBPs, one of which is more complicated and depends on the availability of additional resources. As expected, intentions accounted for much less of the variance in the use of the more complicated and resource-dependent EBP [5, 40]. The intention to use and EBP will not lead to success if the classroom resources needed are unavailable. Teachers who had strong intentions to perform DT may have later found that they lacked the needed materials (which study staff provided for visual schedules but not DT). Their intention may also have been hampered by a chaotic classroom environment and an unfavorable ratio of staff to students. Prediction is also known to decline when the behavior involves a series of actions; performance of the behavior will fail unless each of the action involved is performed [40, 48, 49]. This decline may also help explain the results because DT requires a long sequence of actions. Individuals tend to overestimate the likelihood that they will complete all the actions in this type of sequence [40, 48, 49].

5. The gap between intention and EBP use is malleable and instructive when choosing an implementation strategy. The association between intention and behavior is not uniformly strong [12, 40], and the relative strength provides valuable information the kind of strategies needed to increase implementation [5]. When the strength of the association is not strong because those who intend to use an EBP are not able to succeed, implementation strategies should be designed to overcome or eliminate the moderating factors that stop practitioners from acting on their intentions [5, 6]. If practitioners are already motivated, they may need to receive skills training or access to personnel and other resources needed to perform the EBP [40]. For example, DT requires a favorable student-to-teacher classroom ratio and dedicated space and materials. In under-resourced public schools, these requirements mean that DT is not always feasible. As another example, if intention does not have a strong relationship with subsequent behavior, practitioners may have strong intentions to use an EBP but forget to do so, perhaps because of a chaotic environment or because they perform some other task out of habit [50]. In these cases, a reminder system may be useful [50]. Interventions that encourage individuals to develop detailed plans for achieving their behavioral goal are also frequently successful at bridging this gap [51, 52].

When intention to use an EBP is not strong, a fundamentally different type of implementation strategy will be most effective and efficient. In this scenario, an implementation strategy should instead be designed to strengthen intention. This can be accomplished using financial incentives and non-financial awards that recognize practitioners’ use of EBPs [5, 6]. Scientifically developed motivational messages can also be deployed to strengthen intention [35, 53].

### Limitations and future directions

Several study limitations should be noted that may have biased the results. First, the study was conducted with a sample working in under-funded public schools in a large urban district. Because needed resources are not always available, teachers may find it difficult or impossible to act on intentions to use an EBP [40]. The classrooms studied are often under-staffed and chaotic, which can make it difficult to initiate, or even remember to use some EBPs despite relatively strong intentions to use that EBP [5, 44, 45]. The setting therefore may have biased our observed association between intentions and EBP use towards the null [40, 54].

Second, there was a relatively long period between measuring intentions and EBP use, which increases the chance that the respondents will experience unmeasured change in the strength of their intention [54, 55]. This potential for unmeasured change may also have biased the observed association between intentions and EBP use towards the null. Third, this study used data on EBP performance from only 1 week, which may not represent what teachers usually do.

Other limitations may have inflated the observed association between intentions and EBP performance. Perhaps, chief among these is our reliance on self-reported EBP if study participants consistently reported more EBP use than was accurate. The high variability in this reporting, however, combined with our prior validation of this method, minimizes concerns regarding this limitation [43–45]. Social desirability bias could have also inflated teachers’ reported intentions, in addition to their reported EBP use, which would not inflate their association. Our prior research does not suggest a strong social desirability bias because the teachers did not rate intentions highly for all EBPs [5]. These teachers also frequently report low EBP implementation, which would not be expected in the presence of social desirability bias. To address social desirability bias, we use strategies that have been effective even when studying sexual or other sensitive behaviors [56–59]. For example, teachers knew their responses would be confidential, and we emphasized the importance of accurate data for scientific purposes. Even if self-reporting did bias our point estimates, it is unlikely to explain why some measures of intention performed much better than others.

Ideally, the order of the survey questionnaire items would be randomized, and they were not in our study. In addition, the study examined only two EBPs with one type of practitioner working in a low-resource setting. Therefore, the results may not generalize to other EBPs or teachers. For example, in this study, the training sessions caused the teachers to be at least aware of each EBP studied here. Subsequently, they reported variation in their strength of intention to use each EBP, but if none of the teachers was aware of either EBP, we would not expect this variation. Conceivably, if teachers or other practitioners were not aware of an EBP, they would have consistently weaker intentions to implement it.

In future studies of predictive validity, investigators can adapt the item stems when studying any EBP and other practitioners, such as those employed by health care organizations. Although the present study tested several recommended and commonly used stems, future studies could include additional stems that may contribute to predictive validity. Finally, as implementation science develops a standardized approach to measure intention, it may be useful to document additional psychometric properties.

## Conclusion

This study compared the predictive validity of different, commonly used approaches to measuring intention. We found that items specifying an EBP of interest, rather than referring generally to “evidence-based practices,” had much stronger predictive validity. We also found that three commonly used indicators with different stems are not always interchangeable. A single item with the “I intend to” stem may be optimal for predicting the use of some EBPs. For other EBPs, predictive validity may be substantially increased by using one or two additional items.

Given the study limitations and the preliminary nature of these conclusions, we recognize the need for additional research. However, the current findings support the predictive validity of a highly pragmatic measure of intention that is very brief, sensitive to change, and adaptable to studies of different EBP. As discussed, a standardized measure of intention that is psychometrically strong, particularly regarding predictive validity, can advance implementation science methods and causal model testing, while also empirically identifying the most promising type of implementation strategy.

## Supplementary information

**Additional file 1.** Survey questionnaire example items.

## Data Availability

Requests for access to de-identified data can be sent to the Penn ALACRITY Data Sharing Committee by contacting research coordinator Kelly Zentgraf at zentgraf@upenn.edu, 3535 Market Street, 3^rd^ floor, Philadelphia, PA 19107.

## Abbreviations

DT: Discrete trial training
EBP: Evidence-based practice
