# Supplementary material for: Predicting implementation: comparing validated measures of intention and assessing the role of motivation when designing behavioral interventions
Source: Implement Sci Commun. 2020 Sep 28;1:81. doi: 10.1186/s43058-020-00050-4 (PMC7523324; doi:10.1186/s43058-020-00050-4)
Supplement: Supplementary file 1 — Additional file 1. Survey questionnaire example items. [file 43058_2020_50_MOESM1_ESM.docx]

**Appendix: Survey questionnaire example items**

*An example questionnaire item that references EBP in general:*

**How likely are you to use evidence-based practices?**

| 1 | 2 | 3 | 4 | 5 | 6 | 7 |
| --- | --- | --- | --- | --- | --- | --- |
| Extremely  Unlikely | Unlikely | Somewhat  Unlikely | Uncertain | Somewhat  Likely | Likely | Extremely  Likely |

*An example questionnaire item that references a specific EBP:*

**Think about running discrete trial training (DT) with students at least 3 days a week.**

**How likely are you to do this?**

| 1 | 2 | 3 | 4 | 5 | 6 | 7 |
| --- | --- | --- | --- | --- | --- | --- |
| Extremely  Unlikely | Unlikely | Somewhat  Unlikely | Uncertain | Somewhat  Likely | Likely | Extremely  Likely |

*An example questionnaire item that references another specific EBP:*

**Think about using the individualized student schedules during each transition.**

**How likely are you to do this?**

| 1 | 2 | 3 | 4 | 5 | 6 | 7 |
| --- | --- | --- | --- | --- | --- | --- |
| Extremely  Unlikely | Unlikely | Somewhat  Unlikely | Uncertain | Somewhat  Likely | Likely | Extremely  Likely |

*An example of how the use of an EBP was measured during follow up using log data:*

How often did the teacher use **individual visual schedules** with students in the classroom during the past week?

| **Student** | **Never** | **Few transitions** | **Some transitions** | **Most transitions** | **Every transition** |
| --- | --- | --- | --- | --- | --- |
|  | **0** | **1** | **2** | **3** | **4** |
|  | **0** | **1** | **2** | **3** | **4** |
|  | **0** | **1** | **2** | **3** | **4** |
|  | **0** | **1** | **2** | **3** | **4** |
|  | **0** | **1** | **2** | **3** | **4** |
|  | **0** | **1** | **2** | **3** | **4** |
|  | **0** | **1** | **2** | **3** | **4** |
|  | **0** | **1** | **2** | **3** | **4** |
